# Supplementary material for: Multimodal gradients unify local and global cortical organization
Source: Nat Commun. 2025 Apr 25;16:3911. doi: 10.1038/s41467-025-59177-4 (PMC12032020; doi:10.1038/s41467-025-59177-4)
Supplement: Supplementary file 1 — Supplementary Information [file 41467_2025_59177_MOESM1_ESM.pdf]

Supplementary Information for

Multimodal gradients unify local and global cortical organization

Yezhou Wang[1]*, Nicole Eichert[2], Casey Paquola[3], Raul Rodriguez-Cruces[1], Jordan DeKraker[1], Jessica Royer[1], Donna Gift Cabalo[1], Hans Auer[1], Alexander Ngo[1], Ilana R. Leppert[1], Christine L. Tardif[1, 4], David A. Rudko[1, 4, 5], Robert Leech[6], Katrin Amunts[7, 8], Sofie L. Valk[3, 9], Jonathan Smallwood[10], Alan C. Evans[1], Boris C. Bernhardt[1]*

* Yezhou Wang

Email: yezhou.wang@mail.mcgill.ca

* Boris C. Bernhardt

Email: boris.bernhardt@mcgill.ca

Figures

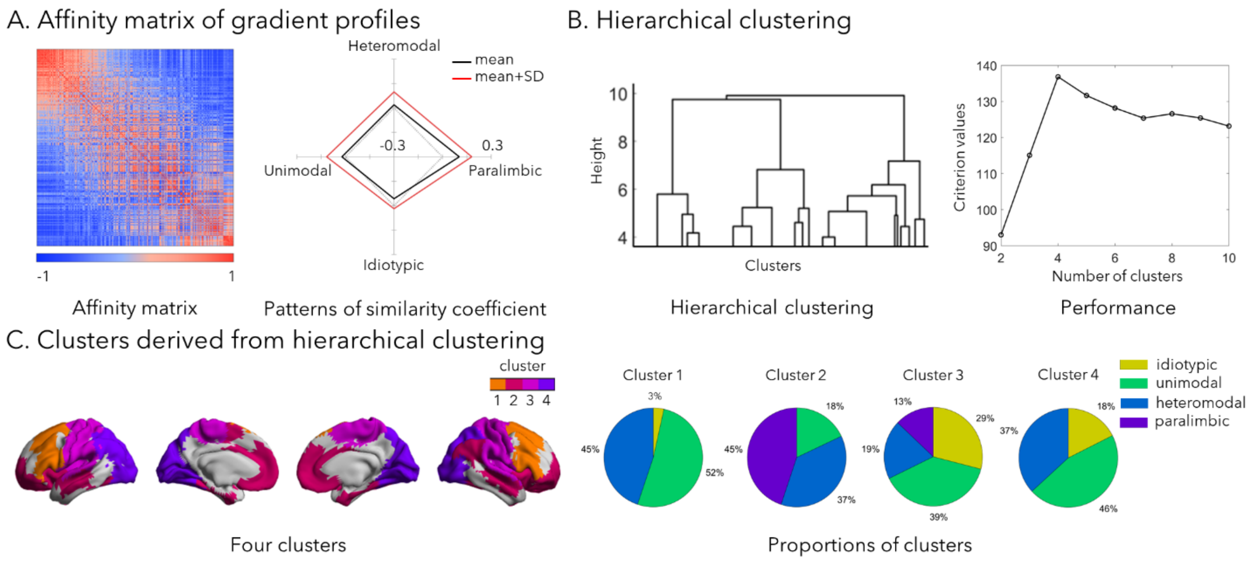

Supplementary Fig. 1. Homogenous patterns of cortical parcels. (A) Affinity matrix of gradient profiles of MPC, SC and FC. We constructed an affinity matrix by calculating the cosine similarity between cortical parcels. The similarity coefficient was determined by averaging each row of the affinity matrix. We examined this coefficient in four cortical hierarchies. **(B) Hierarchical clustering**. We performed hierarchical clustering based on the affinity matrix. The left panel illustrates the clustering results, while the right panel displays the criterion values of hierarchical clustering with different cluster numbers. **(C) Clusters derived from hierarchical clustering**. The left panel showcases the four clusters obtained from hierarchical clustering. The right panel shows the proportions of four cortical hierarchies within each cluster.

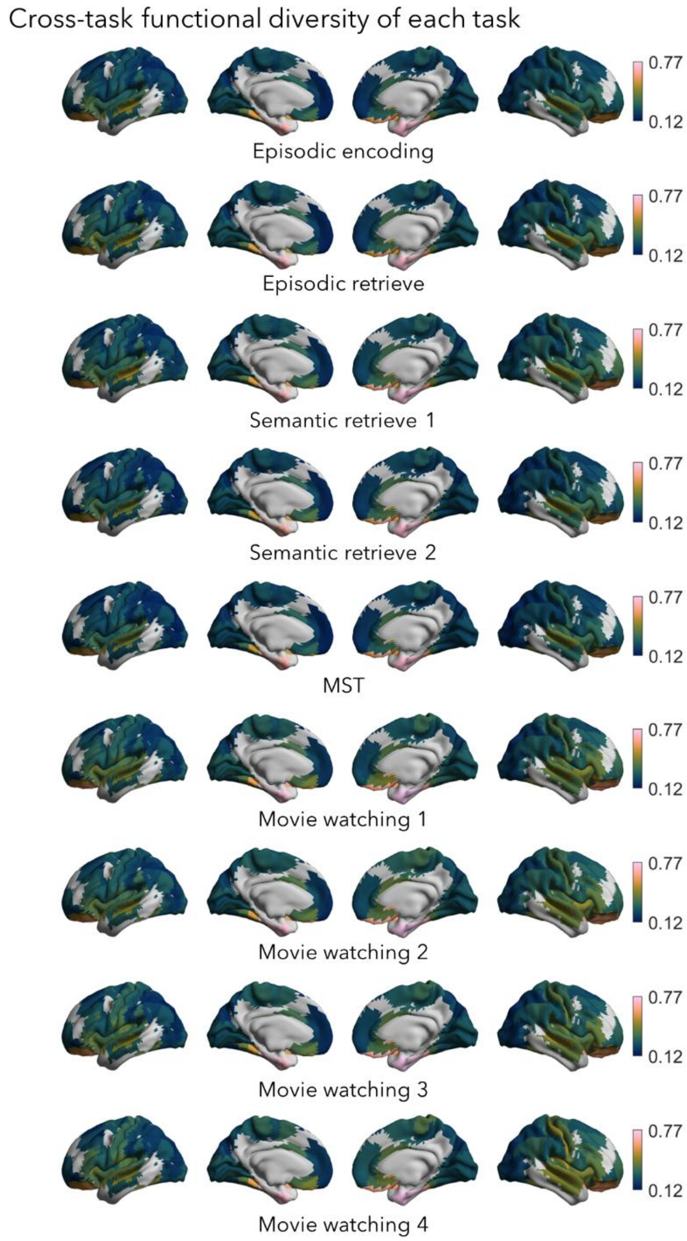

Supplementary Fig. 2. Cross-task functional diversity of each task. The area-wise cross-task diversity matrix shown in the left panel of Fig. 3B was displayed on nine brain maps. All maps share the common color bar and value range.

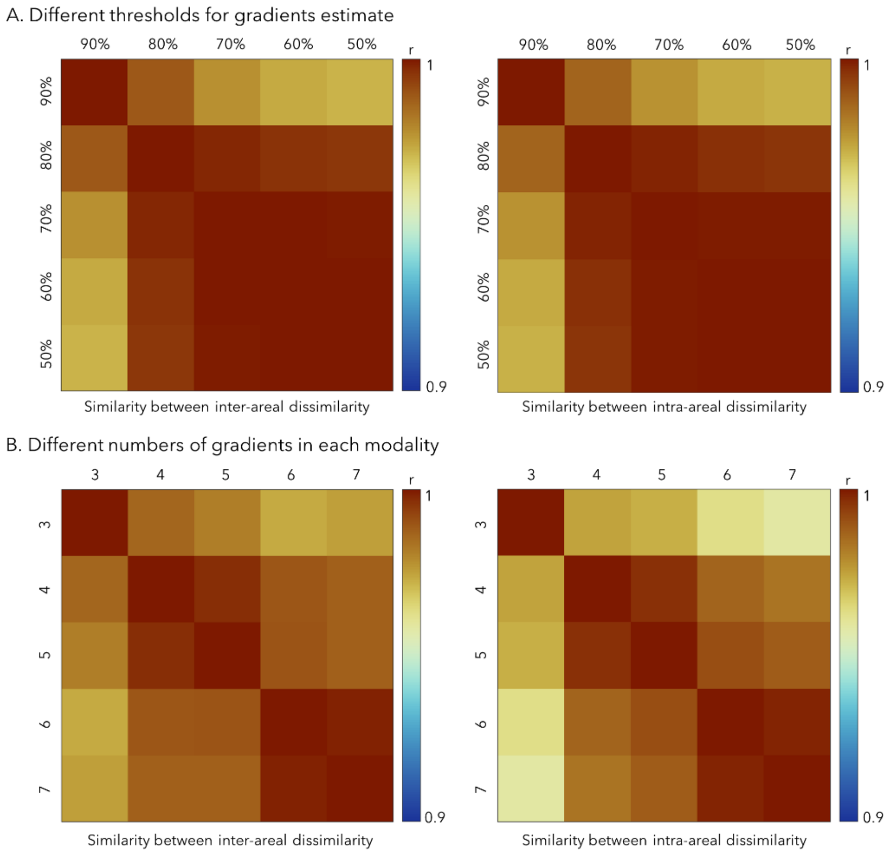

Supplementary Fig. 3. Robustness analysis. (A) Different thresholds for gradients estimate. Multimodal gradients were recalculated using connectivity thresholds ranging from 50% to 90%. Pearson's correlation coefficients were computed between inter/intra-areal dissimilarities derived from multimodal gradients with varying thresholds. **(B) Different numbers of gradients in each modality.** Gradient profiles were recalculated using different numbers of gradients (3-7). Pearson's correlation coefficients were computed between inter/intra-areal dissimilarity derived from gradient profiles estimated with varying numbers of gradients.

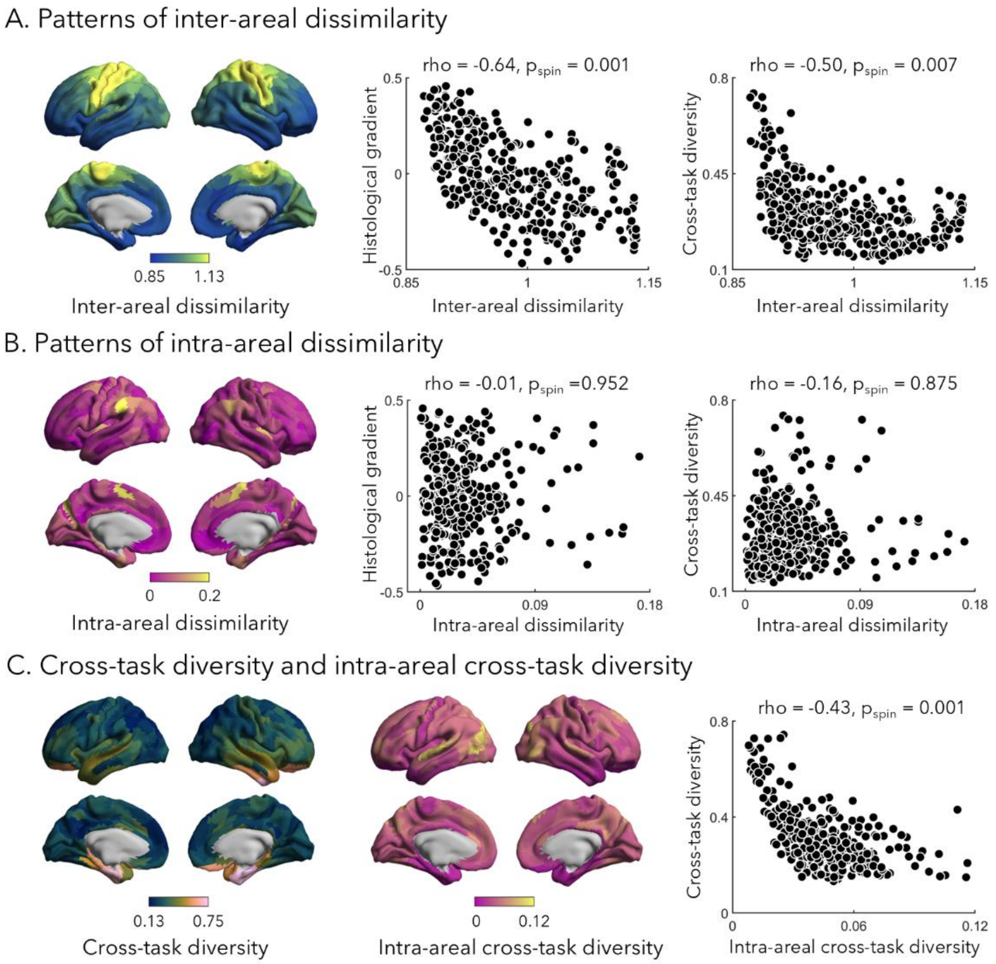

Supplementary Fig. 4. Replication analysis on the Glasser atlas. (A-C) Inter-areal dissimilarity, intra-areal dissimilarity, cross-task diversity, and intra-areal cross-task diversity were estimated using the Glasser atlas (1). Following same methods as the main analysis, spearman's correlation coefficients were computed to examine associations, with p-values corrected using spin permutation tests. For all correlation analyses, sample size n=228. Source data are provided as a Source Data file.

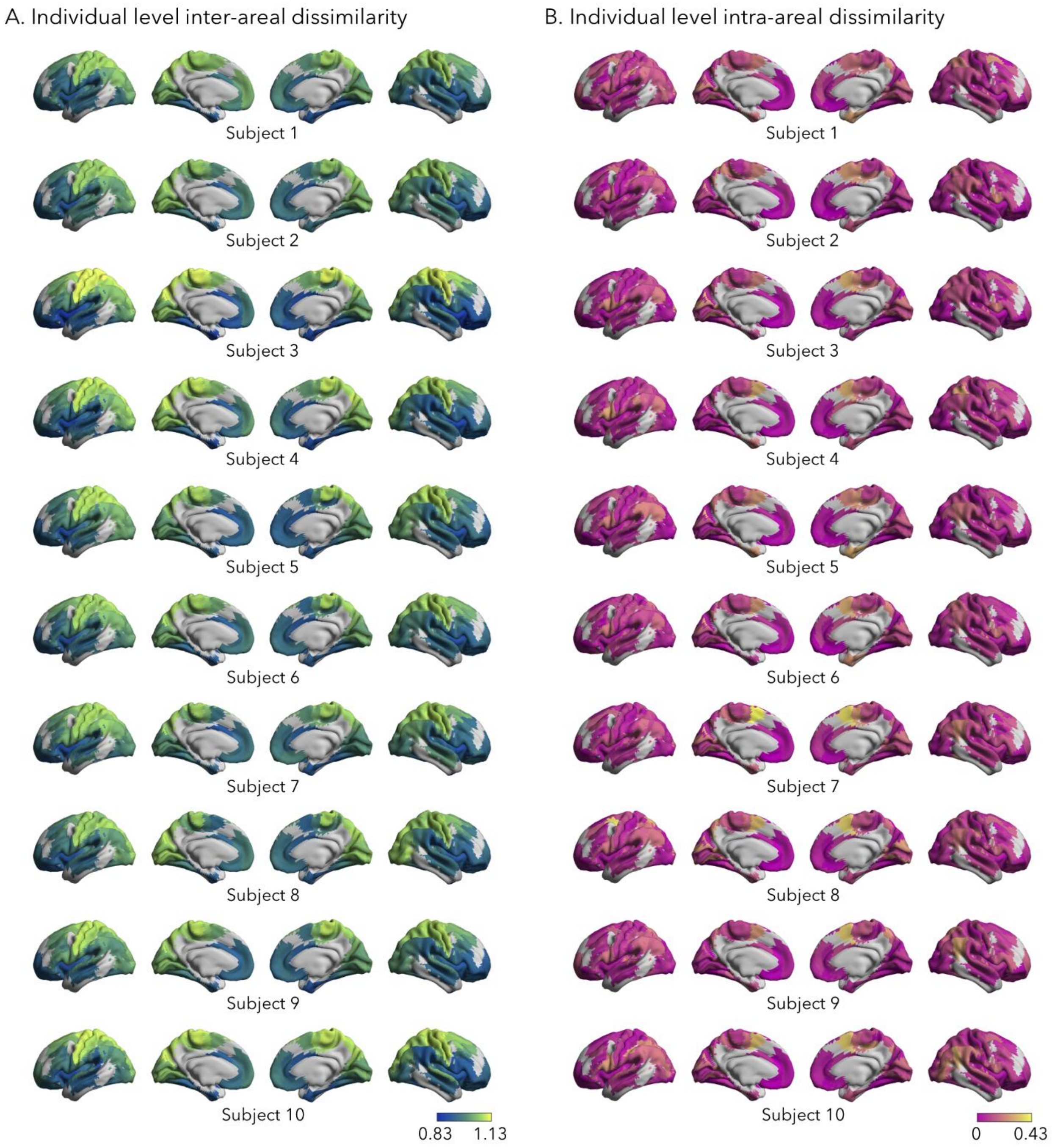

Supplementary Fig. 5. Reliability analysis at the single subject level. (A-B) Individual level inter- and intra-areal dissimilarity of 10 participants in the PNI dataset.

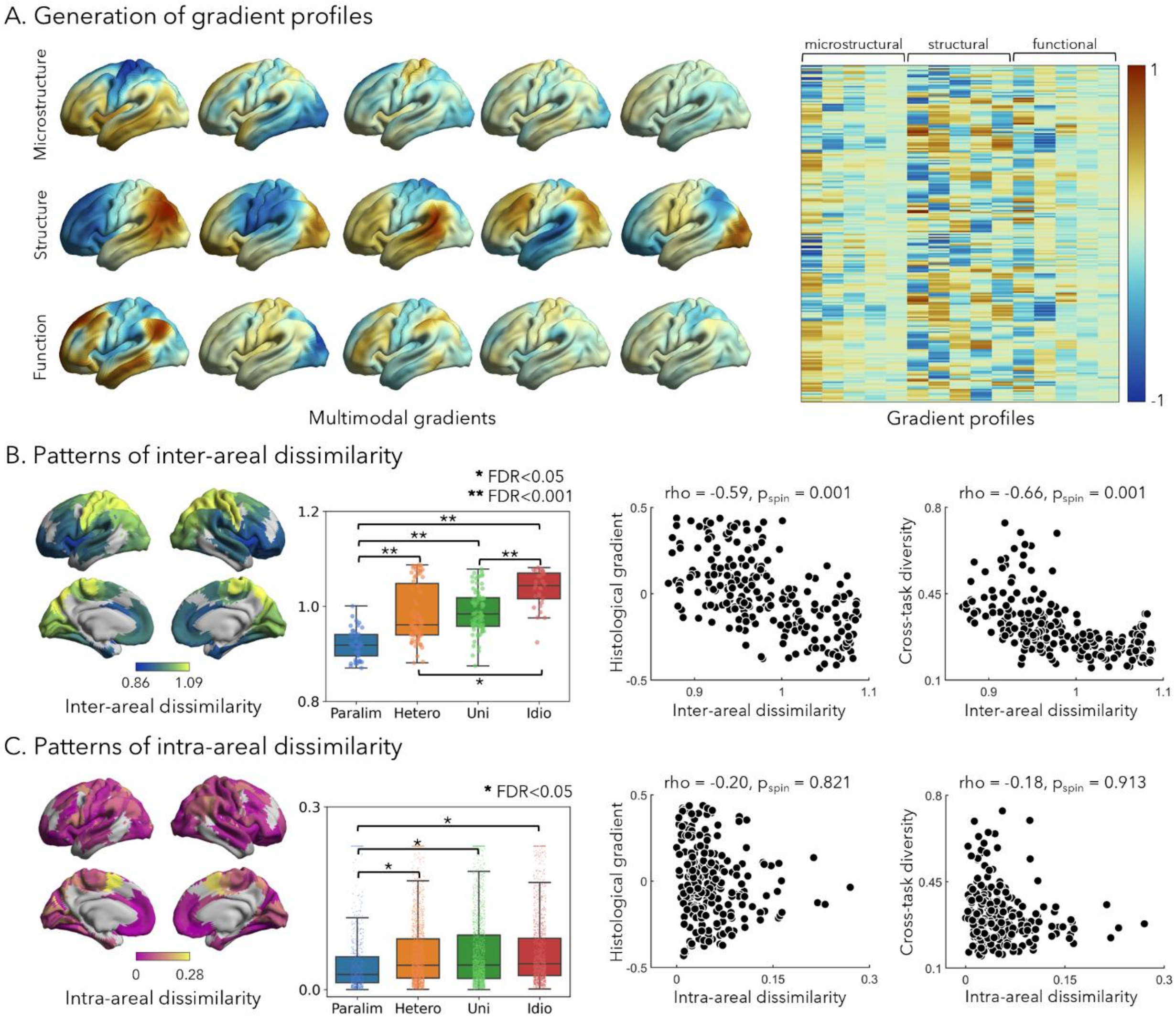

Supplementary Fig. 6. Replication analysis in a dataset scanned at 7T MRI. (A-C) The generation of multimodal gradients and gradient profiles and the assessments of associations between inter-areal and intra-areal dissimilarity followed similar methods as the main analysis. Box plots display the median (center line), interquartile range (box bounds = 25th to 75th percentiles), and whiskers extending to the minimum and maximum values within 1.5× the interquartile range (IQR). Bar charts in Panel B: Sample sizes: Paralim (45), Hetero (75), Uni (79), and Idio (29); all are technical replicates. Bar charts in Panel C: Sample sizes: Paralim (789), Hetero (2,342), Uni (2,403), and Idio (1,779); all are technical replicates. Box plots display the median (center line), interquartile range (box bounds = 25th to 75th percentiles), and whiskers extending to the minimum and maximum values within 1.5× the IQR. Bars are color-coded as follows: blue for paralimbic, orange for heteromodal, green for unimodal, and red for idiotypic. For all correlation analyses, sample size n=228. Source data are provided as a Source Data file. Abbreviation: Paralim: Paralimbic; Hetero: Heteromodal; Uni: Unimodal; Idio: Idiotypic.

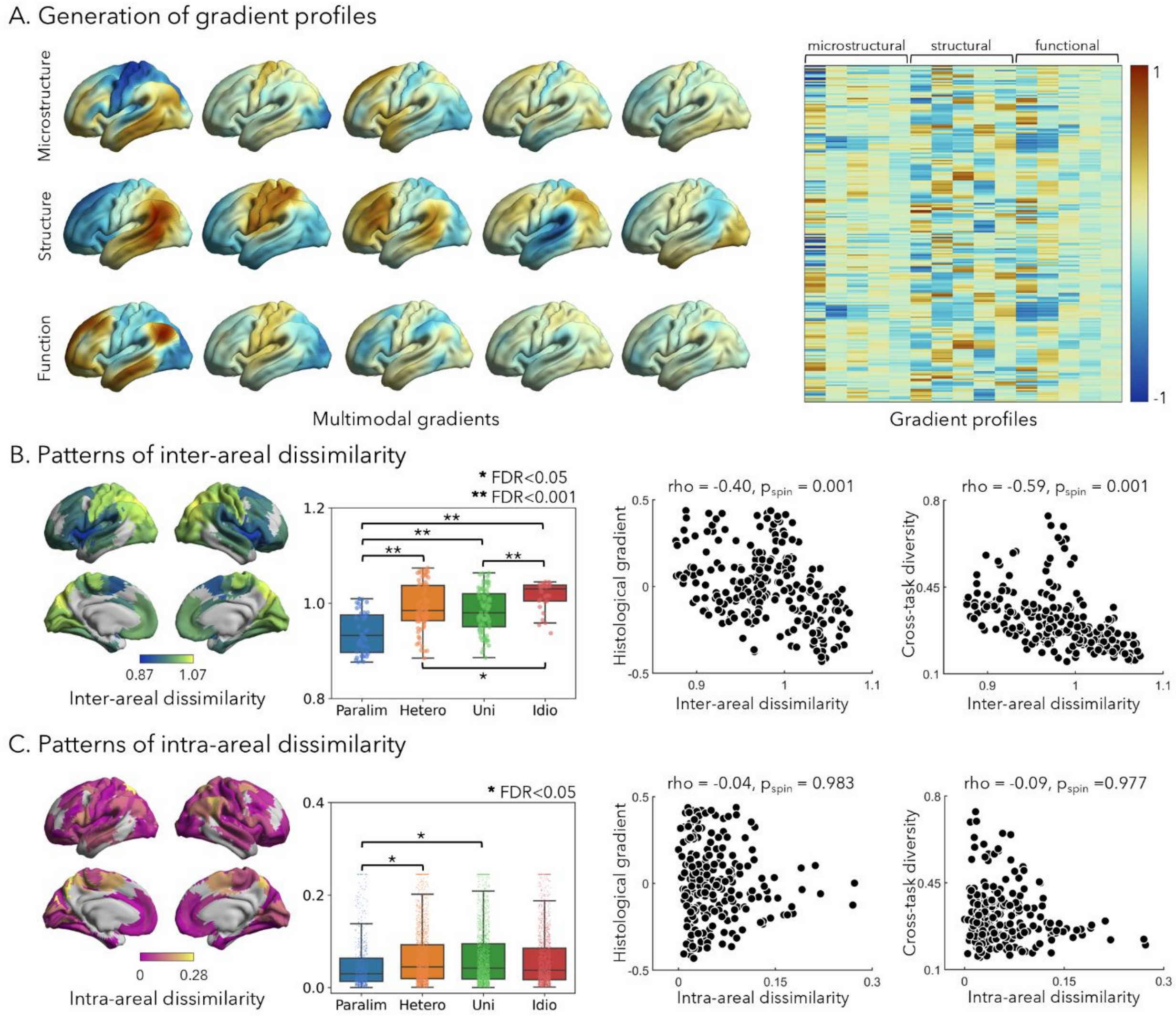

Supplementary Fig. 7. Replication analysis in an independent dataset scanned at 3T MRI. (A-C) The generation of multimodal gradients and gradient profiles and the assessments of associations between global and intra-areal dissimilarity followed similar methods as the main analysis. Box plots display the median (center line), interquartile range (box bounds = 25th to 75th percentiles), and whiskers extending to the minimum and maximum values within 1.5× the IQR. Panel B: Sample sizes: Paralim (45), Hetero (75), Uni (79), and Idio (29); all are technical replicates. Box plots display the median (center line), interquartile range (box bounds = 25th to 75th percentiles), and whiskers extending to the minimum and maximum values within 1.5× the IQR. Bars are color-coded as follows: blue for paralimbic, orange for heteromodal, green for unimodal, and red for idiotypic. For all correlation analyses, sample size n=228. Source data are provided as a Source Data file. Abbreviation: Paralim: Paralimbic; Hetero: Heteromodal; Uni: Unimodal; Idio: Idiotypic.

Supplemental Table 1.

	Correlations with inter-aeal dissimilarity		Partial correlations between inter-aeal dissimilarity and cross-task diversity	
	r	p_{spin}	r	p_{spin}
B1+ field	-0.05	0.22	-0.71	<0.001
T1 CNR	0.12	0.28	-0.70	<0.001
DWI SNR	-0.11	0.34	-0.71	<0.001
fMRI tSNR	0.66	<0.001	-0.46	<0.001

Pearson's correlation coefficients were calculated between inter-areal dissimilarity and each of the following imaging metrics separately: B1+ field, T1 CNR, DWI SNR, and fMRI tSNR. Additionally, partial correlations were computed between inter-areal dissimilarity and cross-task diversity while controlling for each of these imaging metrics separately. To account for spatial autocorrelation, a spin test was performed. False discovery rate (FDR) correction was applied to adjust for multiple comparisons. For all correlation analyses, sample size n=228. All correlations were two-sided.

Supplemental References

1. M. F. Glasser *et al.*, A multi-modal parcellation of human cerebral cortex. *Nature* **536**, 171-178 (2016).
