# Supplementary material for: Multimodal gradients unify local and global cortical organization
Source: Nat Commun. 2025 Apr 25;16:3911. doi: 10.1038/s41467-025-59177-4 (PMC12032020; doi:10.1038/s41467-025-59177-4)
Supplement: Supplementary file 2 — Reporting Summary [file 41467_2025_59177_MOESM2_ESM.pdf]

nature portfolio

Corresponding author(s): Yezhou Wang
Boris Bernhardt

Last updated by author(s): Feb 24, 2025

Reporting Summary

Nature Portfolio wishes to improve the reproducibility of the work that we publish. This form provides structure for consistency and transparency in reporting. For further information on Nature Portfolio policies, see our Editorial Policies and the Editorial Policy Checklist.

Statistics

For all statistical analyses, confirm that the following items are present in the figure legend, table legend, main text, or Methods section.

n/a	Confirmed	
☐	☒	The exact sample size (*n*) for each experimental group/condition, given as a discrete number and unit of measurement
☐	☒	A statement on whether measurements were taken from distinct samples or whether the same sample was measured repeatedly
☐	☒	The statistical test(s) used AND whether they are one- or two-sided<br>*Only common tests should be described solely by name; describe more complex techniques in the Methods section.*
☐	☒	A description of all covariates tested
☐	☒	A description of any assumptions or corrections, such as tests of normality and adjustment for multiple comparisons
☐	☒	A full description of the statistical parameters including central tendency (e.g. means) or other basic estimates (e.g. regression coefficient) AND variation (e.g. standard deviation) or associated estimates of uncertainty (e.g. confidence intervals)
☐	☒	For null hypothesis testing, the test statistic (e.g. *F*, *t*, *r*) with confidence intervals, effect sizes, degrees of freedom and *P* value noted<br>*Give P values as exact values whenever suitable.*
☒	☐	For Bayesian analysis, information on the choice of priors and Markov chain Monte Carlo settings
☒	☐	For hierarchical and complex designs, identification of the appropriate level for tests and full reporting of outcomes
☐	☒	Estimates of effect sizes (e.g. Cohen's *d*, Pearson's *r*), indicating how they were calculated

Our web collection on statistics for biologists contains articles on many of the points above.

Software and code

Policy information about availability of computer code

Data collection	The main MRI data were collected on 3T and 7T Siemens scanners.
Data analysis	Micapipe (v0.2.3) was used for multimodal MRI processing. BrainSpace (v0.1.10) was used to derive macroscale gradients. Custom Matlab code was used for the main analysis, which is openly available on https://github.com/MICA-MNI/Wang_MultimodalGradient

For manuscripts utilizing custom algorithms or software that are central to the research but not yet described in published literature, software must be made available to editors and reviewers. We strongly encourage code deposition in a community repository (e.g. GitHub). See the Nature Portfolio guidelines for submitting code & software for further information.

Data

Policy information about availability of data

All manuscripts must include a data availability statement. This statement should provide the following information, where applicable:

- Accession codes, unique identifiers, or web links for publicly available datasets
- A description of any restrictions on data availability
- For clinical datasets or third party data, please ensure that the statement adheres to our policy

The MRI data of the 7T discovery dataset is openly available at the OSF platform (https://osf.io/mhq3f/). The Julich-Brain atlas is available at the EBRAINS platform (https://www.ebrains.eu/tools/human-brain-atlas). The 3T MICA-MICs replication data is openly available at https://portal.conp.ca/dataset?id=projects/mica-mics.

Research involving human participants, their data, or biological material

Policy information about studies with human participants or human data. See also policy information about sex, gender (identity/presentation), and sexual orientation and race, ethnicity and racism.

Reporting on sex and gender	We collected similar proportions of males and females in the discovery 7T dataset (5 males, 5 females), the validation 7T dataset (5 males, 7 females), and the validation 3T dataset (53 males, 47 females). Biological sex was determined by self-reporting. No self-reported gender was acquired. We have also verified consistency of our main effects when performing sex-disaggregated analyses. Sample size was too low to compare between sexes directly.
Reporting on race, ethnicity, or other socially relevant groupings	There were no socially constructed nor socially relevant categorization variables used in this manuscript.
Population characteristics	The participants in this study are healthy young adults. 10 unrelated healthy adults (age: 29.20±5.20 years, 5 females).
Recruitment	Participants reached out to us after seeing our advertisements or promotional materials. We then selected qualified individuals to take part in the experiment based on our design criteria.
Ethics oversight	The studies were approved by the Ethics Committees of McGill University and the Montreal Neurological Institute and Hospital, respectively.

Note that full information on the approval of the study protocol must also be provided in the manuscript.

Field-specific reporting

Please select the one below that is the best fit for your research. If you are not sure, read the appropriate sections before making your selection.

☒ Life sciences ☐ Behavioural & social sciences ☐ Ecological, evolutionary & environmental sciences

For a reference copy of the document with all sections, see nature.com/documents/nr-reporting-summary-flat.pdf

Life sciences study design

All studies must disclose on these points even when the disclosure is negative.

Sample size	To improve data quality, we collected MRI data from each participant on three separate days. We took into account time costs and feasibility when determining the sample size for the study.
Data exclusions	No data were excluded form the analysis.
Replication	We conducted replication analysis based on 12 healthy young adults with one session scanned at 7T, and a second replication analysis in an independent sample of 100 healthy adults scanned at 3T. All attempts at replication were successful.
Randomization	There is only one group in this study.
Blinding	We did not group participants in this study.

Reporting for specific materials, systems and methods

We require information from authors about some types of materials, experimental systems and methods used in many studies. Here, indicate whether each material, system or method listed is relevant to your study. If you are not sure if a list item applies to your research, read the appropriate section before selecting a response.

Materials & experimental systems

n/a	Involved in the study	
☒	☐	Antibodies
☒	☐	Eukaryotic cell lines
☒	☐	Palaeontology and archaeology
☒	☐	Animals and other organisms
☒	☐	Clinical data
☒	☐	Dual use research of concern
☒	☐	Plants

Methods

n/a	Involved in the study	
☒	☐	ChIP-seq
☒	☐	Flow cytometry
☐	☒	MRI-based neuroimaging

Plants

Seed stocks

We did not use any seeds or plants in this study.

Novel plant genotypes

We did not use any seeds or plants in this study.

Authentication

We did not use any seeds or plants in this study.

Magnetic resonance imaging

Experimental design

Design type

Multimodal MRI, including non-task and task-related imaging.

Design specifications

N/A

Behavioral performance measures

N/A

Acquisition

Imaging type(s)

Functional, structural and diffusion MRI

Field strength

7T and 3T

Sequence & imaging parameters

7T MRI data: spin-echo; EPI; FOV=224×224; matrix=320×320; slice thickness=1.1 mm (DWI); slice thickness=1.9 mm (fMRI). For MP2RAGE, TR=5170 ms, TE=2.44 ms, flip angle=4°. For DWI, TR=7383 ms, TE=70.60 ms, flip angle=90°, refocusing flip angle=180°. For fMRI, TR=1690 ms, TE1=10.8 ms, TE2=27.3 ms, TE3=43.8 ms, flip angle=67°.

Area of acquisition

A whole brain scan was used.

Diffusion MRI ☒ Used ☐ Not used

Parameters

DWI data was acquired using a multiband accelerated 2D spin-echo echo-planar imaging sequence. The acquisition included three shells with b-values of 300, 700, and 2000 s/mm2, and 10, 40, and 90 diffusion weighting directions per shell, respectively. The parameters used were: 1.1 mm isotropic voxels, TR=7383 ms, TE=70.60 ms, flip angle=90°, refocusing flip angle=180°, FOV=224×224 mm2, slice thickness=1.1 mm, multi-band factor=2.

Preprocessing

Preprocessing software

MRI data preprocessing is finished using Micapipe v0.2.3, which is available at https://github.com/MICA-MNI/micapipe . By using Micapipe, several softwares were called upon to complete the data preprocessing. MP2RAGE scans of each subject were reoriented using FSL, linearly co-registered, averaged, with background noise removed, corrected for intensity nonuniformity using N4 bias field correction from ANTS, and segmented into white and grey matter using FSL FAST. Resulting volumes were skull stripped using FSL. Cortical surface models were generated from native T1w scans using FastSurfer. Regarding the DWI data, pre-processing was carried out using MRtrix in the native DWI space. The DWI data underwent denoising, b0 intensity normalization, and correction for susceptibility distortion, head motion, and eddy currents. These corrections were performed using FSL and involved utilizing two b=0 s/mm2 volumes with reverse phase encoding. Anatomical masks for tractography were non-linearly co-registered to native DWI space using the deformable SyN approach implemented in ANTs. For the rs-fMRI scans, pre-processing steps were conducted using AFNI and FSL tools. The first five volumes were discarded to ensure magnetic field saturation. The volume timeseries were registered to FastSurfer space using boundary-based registration implemented in ANTs using linear and non-linear methods.

Normalization

Data was normalized using linear methods.

Normalization template

MNI152

Noise and artifact removal

The MP2RAGE scans of each subject were reoriented using FSL, linearly co-registered, averaged, with background noise removed, corrected for intensity nonuniformity using N4 bias field correction from ANTS, and segmented into white and grey matter using FSL FAST. For fMRI data, we applied FMRIB's ICA-based X-noiseifier (ICA-FIX) and spike regression to remove timepoints with large motion spikes, effectively removing nuisance signals. The DWI data underwent denoising b0 intensity normalization, and correction for susceptibility distortion, head motion, and eddy currents.

Volume censoring

The volumes were visually inspected by an experienced neurologist. The abnormal data were excluded.

Statistical modeling & inference

Model type and settings: multivariate; fixed effects

Effect(s) tested: Effect of task was not estimated in this study; ANOVA is not used.

Specify type of analysis: ☒ Whole brain ☐ ROI-based ☐ Both

Statistic type for inference (See Eklund et al. 2016): Vertex-wise gradients were estimated. Areal-wise gradient profiles were computed. The Glasser-360 atlas with 360 regions was used in this study.

Correction: The resulting p-values were corrected for spatial autocorrelations using 1,000 spin permutation tests. FDR corrections were applied to correct for multiple comparisons.

Models & analysis

n/a	Involved in the study
☐	☒ Functional and/or effective connectivity
☐	☒ Graph analysis
☐	☒ Multivariate modeling or predictive analysis

Functional and/or effective connectivity: Pearson correlation coefficients between vertex-wise time series were calculated to generate the functional connectivity.

Graph analysis: We estimated participation coefficient for the subjects in this study.

Multivariate modeling and predictive analysis: Dimensionality reduction techniques (PCA, diffusion map embedding) were used in this study.
